# Transcriptome analysis and annotation: SNPs identified from single copy annotated unigenes of three polyploid blueberry crops

**DOI:** 10.1371/journal.pone.0216299

**Published:** 2019-04-29

**Authors:** Yunsheng Wang, Muhammad Qasim Shahid, Fozia Ghouri, Sezai Ercişli, Faheem Shehzad Baloch, Fei Nie

**Affiliations:** 1 College of Life and Health Science, Kaili University, Kaili City, Guizhou Province, China; 2 State Key Laboratory for Conservation and Utilization of Subtropical Agro-Bioresources, South China Agricultural University, Guangzhou, China; 3 College of Agriculture, South China Agricultural University, Guangzhou, Guangdong Province, China; 4 Department of Horticulture, Faculty of Agriculture, Ataturk University, Erzurum, Turkey; 5 Department of Field Crops, Faculty of Agricultural and Natural Sciences, Abant İzzet Baysal University, Bolu, Turkey; 6 Biological Institute of Guizhou Province, Guiyang City, Guizhou Province, China; Institute for Horticultural Plants, China Agricultural University, CHINA

## Abstract

Blueberry is a kind of new rising popular perennial fruit with high healthful quality. It is of utmost importance to develop new blueberry varieties for different climatic zones to satisfy the demand of people in the world. Molecular marker assisted breeding is believed to be an ideal method for the development of new blueberry varieties for its shorter breeding cycle than the conventional breeding. Simple sequence repeats (SSRs) and single nucleotide polymorphisms (SNPs) markers are widely used molecular tools for marker assisted breeding, which could be detected at large scale by the transcriptome sequencing. Here, we sequenced the leaves transcriptome of 19 rabbiteye (*Vaccinium ashei* Reade), 13 southern highbush (*Vaccinium*. *corymbosum* L × native southern *Vaccinium Spp*) and 22 cultivars of northern highbush blueberry (*Vaccinium corymbosum* L) by using next generation sequencing technologies. A total of 80.825 Gb clean data with an average of about 12.525 million reads per cultivar were obtained. We assembled 58,968, 55,973 and 53,887 unigenes by using the clean data from rabbiteye, southern highbush and northern highbush blueberry cultivars, respectively. Among these unigenes, 3599, 3495 and 3513 unigenes were detected as candidate resistance genes in three blueberry crops. Moreover, we identified more than 8756, 9020, and 9198 SSR markers from these unigenes, and 7665, 4861, 13,063 SNPs from the annotated single copy unigenes, respectively. The results will be helpful for the molecular genetics and association analysis of blueberry and the basic molecular information of pest and disease resistance of blueberry, and would also offer huge number of molecular tools for the marker assisted breeding to produce blueberry cultivars with different adaptive characteristics.

## Introduction

Blueberry is perennial flowering shrub or small tree, which comprises of about twenty members that belong to section) *Cyanococcus*, genus *Vaccinium*, and family Ericaceae [1]. The blueberry is a delicious fruit, and its fruits are famous in the world for its high anthocyanins contents, and it is listed among the top five healthful fruits (non-citrus) in North America [2,3]. The previous studies showed that blueberry anthocyanins have multiple healthful functions including retarding age-related diseases like Alzheimer’s and enhancing memory [4], reducing eye strain, preventing macular degeneration, exhibiting anti-cancer activity [5,6], and reduce the risk of heart diseases [7]. Blueberry fruit is also a good raw material of sauces, juices and wine [8,9], and used as a dye because of high pigment contents [10].

In the recent decade, the blueberry production has increased significantly in the world, especially the production of new emerging countries from Asia, Oceania and South America [11–13]. The world production of highbush blueberry, which is a major blueberry crop, had passed the 1-billion pound in 2012 [14]. However, blueberry cultivars planted in the whole world are still mainly from North America [15], and the new blueberry producing countries have different climatic and soil conditions compared to the native blueberry producing area [16]. In order to cope with the challenges from various ecological and climatic conditions, more new widely adaptive cultivars are required for the development and growth of blueberry industry. However, blueberry is a perennial fruit crop with long juvenile period and complex ploidy genome [17–19]. Therefore, it required a long time to overcome these unfavorable factors and to select key traits in the breeding procession by conventional methods [20–22], and also needs to spend a lot of manpower and resources [23]. Modern molecular marker assisted breeding techniques and genetic engineering techniques are apt to overcome these problems and accelerate the breeding process [19].

With the advent of high-throughput sequencing technology and the development of bioinformatics analysis, genomics research has become a common method for biological laboratories. Blueberry research has entered into the genomic era with the availability of huge genomic data [24]. For example, the molecular mechanism of the cold adaptation of blueberry was studied extensively by using functional genomics methods, especially RNA-seq sequencing technique, and the gene expression analysis under the cold environment [25–31]. The metabolic related genes of blueberry antioxidant substances were explored by transcriptome analysis [32]. The changes in gene expression profiles of blueberry after infection with *Bacillus anthracis* were studied by RNA-seq technique [33]. Metabolite profiling showed transcriptional regulation of abscisic acid and flavonoids metabolism during the development of blueberry fruit [34], and candidate genes involved in fruit ripening were identified [35]. The EST sequence database of cultured blueberries (*Vaccinium corymbosum*) was also established in 2007 [36]. Meanwhile, a reference genome of blueberry (*Vaccinium corymbosum* with diploid genome) has been published, and researchers can access it by the genome Browser8.5.2 software (http://bioviz.org/igb/). However, the above studies were only limited to an individual blueberry cultivar, and the genome information about different blueberry cultivars or populations have not been reported yet. Moreover, there are few studies about the exploitation of SSR or SNP markers and haplotype-phased genome assembly of blueberry by genotyping by sequencing (GBS) and whole genome sequencing [37–39].

Molecular markers are indispensable tools for marker assisted breeding. The SSR and SNP markers are two attractive and widely used because of many merits including co-dominant, reproducibility, locus-specificity, and random genome-wide distribution in many organisms [40,41]. In this genomic era, the development of SSR and SNP markers by high-throughput next-generation sequencing platform has been popular work and marker assisted breeding has also entered into the genomics era [42–45]. In the present study, we sequenced the leaves transcriptome of 19 rabbiteye blueberry cultivars, 13 southern highbush blueberry cultivars and 22 cultivars of northern highbush blueberry by using next generation sequencing technologies. Our aims were (1) to collect functional genome information about different blueberry cultivars; (2) to uncover the preliminary molecular mechanism of blueberry adaptation by mining resistance genes; and (3) to develop SSR and SNP markers to assist in the breeding and other corresponding studies about blueberry.

## Materials and methods

### Ethics statement

No specific permissions were required for these locations/activities because all samples were collected from blueberry germplasm nursery of Majiang Blueberry Industry Engineering Technology Center, Guizhou, China. We collected leaves from blueberry cultivars for research, and also confirmed that the field studies did not involve any endangered or protected species.

### Plant material and RNA extraction

We extracted the total RNA from the young leaves of 2–3 years old seedlings of 54 blueberry cultivars that were planted at blueberry germplasm nursery of Majiang Blueberry Industry Engineering Technology Center (Wuyangma village, Xuanwei town, Majiang county, Guizhou province, China), including 19 rabbiteye, 22 northern highbush and 13 southern highbush blueberry cultivars (S1 Table). The total RNA from young leaves of all cultivars was extracted by using the Spectrum plant total RNA kit (Sigma-Aldrich-STRN250 MSDS, USA) and strictly followed the guidelines provided by the company. High quality RNA with RIN (RNA integrity number) above 7.0 was used for RNA sequencing.

### Library construction and sequencing

High quality total extracted RNAs (A260/A230 of OD value more than 2.0, A260/A280 OD value between 1.8–2.0, electrophoretic bands clear, concentration more than 50ng/μL) were used to construct the paired-end sequencing libraries, and the sequencing was done according to the sequencer provider’s instructions as follow: First, the total RNA was treated with DNAse and then separated poly-A-containing mRNA from the total RNA by using poly-T-oligo-attached magnetic beads. Second, the purified mRNA sequences were fragmented into approximately 300~500 base length fragments, and these mRNA fragments were used as template to synthetize the first single strand of cDNA, and then the first strand of cDNA was used as template to synthetize the second strand of cDNA. Third, the synthetized double strands were purified and quantified after carrying out the reaction of end repair, A-tailing and adapter ligation. Then the purified cDNA was enriched by a 15-cycle-PCR reaction to complete sequencing library. Finally, paired-end sequencing was conducted on Illumina HighSeq 4000 platform. Raw reads with fastq format have been deposited to NCBI and are available at genbank with ID: PRJNA511922.

### Raw data filtering

We obtained the clean reads for further assembly by filtering the raw reads based on the following steps and rules: 1) removing reads containing adapters; 2) removing reads containing more than 10% of unknown nucleotides (N); 3) removing reads containing more than 50% of low quality (Q-value≤20) bases.

### De novo assembly

Though the genome of a highbush diploid blueberry is available (http://bioviz.org/igb/), but the sequencing coverage and the genome integrity of reference genome is very low. So we assembled the unigenes of three kinds of blueberry crops independently by using program “Trinity”, a software package designed specifically for the assembling of short reads without reference genome [46]. The unigenes with a length longer than 201 bp were accounted for statistics and used for further analysis.

### Annotation of unigenes

We executed basic annotations including protein functional annotation, pathway annotation, COG/KOG functional annotation and Gene Ontology (GO) enrichment analysis to predict the molecular functions of assembled unigenes. First, we used BLASTx program [47] with an E-value threshold of 1e-5 to hit against the NCBI non-redundant protein database (http://www.ncbi.nlm.nih.gov), the Swiss-Port protein database (http://www.expasy.ch/sprot), the Kyoto Encyclopedia of Genes and Genomes (KEGG) database [48], and the COG/KOG database [49]. We obtained the protein functional annotation codes of corresponding unigenes according to the best alignment results. Then we performed GO functional annotation of unigenes by using the Blast2GO software [50], and the functional classification of unigenes was done using WEGO software [51].

### Identification of resistance genes

We used all assembled unigenes to query the plant resistance genes database (PRGdb; http://prgdb.org) with an E-value threshold of 1e-5.

### Detection of SSR markers and primer designing

We used program MISA (http://pgrc.ipk-gatersleben.de/misa/) to identify SSR markers and designed corresponding primers by using following parameters: (1) motif ranged from 2 to 6 nucleotides; (2) minimum repeat units were six for 2 nucleotide repeat motifs, five for 3 nucleotide repeat motifs, four for 4–6 nucleotide repeat motifs; (3) the maximum interruption length between two SSR markers was set as 100 bp. The program Primer 3 (http://primer3.ut.ee/) was used to design primers with the following criteria: The GC contents of primer sequences were ranged from 40% to 60%, and the size of expected PCR product was ranged from 100 to 250 bp.

### SNP calling

We used program tophat v2.0.14 which is built in bowtie software package (http://bowtie-bio.sourceforge.net/index.shtml) to call the original SNPs dataset by setting default parameters. To avoid the false positive mutant loci as much as possible, we filtered the original SNP dataset by following criteria: sequencing quality of SNP loci base reach to Q30, the read depth of opposite base of SNP loci reach to five, minor allele frequency of SNP loci greater than 15%, and SNP found only in annotated single copy unigenes. To identify single copy unigenes, we first executed two-two alignment of all unigenes that belong to different species by using blastp method, and the unigene pairs with E-value lower than 1e-7 of were regarded as homologous genes, and then we clustered unigenes that are homologous to each other into one gene family by running the program of OrthoMCL (http://orthomcl.org/orthomcl/). If a gene family includes only one unigene in each species, then it was regarded as a single copy unigene.

## Results

### Data statistics and Unigenes assembly

We obtained about 248.26, 139.28, 288.81 million raw reads from leaves transcriptome of 19 rabbiteye, 13 southern highbush, and 22 northern highbush blueberry cultivars by using HighSeq 4000 platform, respectively. After filtering the reads containing adapters, more than 10% of unknown nucleotides and low quality bases (<Q20), 246.84, 138.47 and 286.89 million clean reads with an average of 12.99, 10.65 and 13.04 million clean reads per cultivar were generated (S1 Table). The clean reads were assembled into 45,535, 42,914 and 43,630 unigenes in rabbiteye, southern highbush and northern highbush blueberry cultivars, and the average length of three unigenes clusters were 857 bp, 873 bp and 896 bp, respectively (S2 Table).

### Annotation of Unigenes

Of the 45,535, 42,914 and 43,630 unigenes, a total of 28,091, 28,115, 27,256 unigenes were functionally annotated by one or more databases, such as Nr, Swiss-Port, KOG and KEGG, which accounted for 61.69%, 65.51% and 62.47% of total unigenes, respectively (Table 1). Among the three kinds of unigenes annotated by Nr database, the top 15 species hit by about 60% annotated unigenes were *Vitis vinifera*, *Theobroma cacao*, *Sesamum indicum*, *Nelumbo nucifera*, *Jatropha curcas*, *Prunus mume*, *Nicotiana tomentosiformis*, *Gossypium arboreum*, *Nicotiana sylvestris*, *Populus euphratica*, *Brassica napus*, *Citrus sinensis*, *Medicago truncatula*, *Solanum tuberosum*, and *Gossypium raimondii* (S3 Table). Among the unigenes annotated by Swiss-Port database, the numbers that fall within the E-value scope of 0~1E150, 1E150~1E125, 1E125~1E100, 1E100~1E75, 1E75~1E50, 1E50~1E25 and 1E25~1E5 based on the match degree were 3866, 929, 949, 1176, 1480, 2155 in terms of rabbiteye blueberry, 3670, 6285; 3828, 958, 912, 1220, 1529, 2192, 3815, 6299 in terms of southern highbush blueberry and 3952, 966, 951, 1171, 1498, 2100, 3512, 5934 in terms of northern highbush blueberry, respectively (S4 Table). Annotation by KOG database showed that most of the unigenes in three kinds of blueberries were involved into “General function prediction only”, and reached to 6204 (36.36%), 6154 (35.75%) and 5990 (36.15%), followed by the molecular function of “signal transduction mechanisms” and “posttranslational modification, protein turnover, chaperones”, and the number reached to 3451 (20.23%), 3375 (19.61%), 3311 (19.98%) and 3238 (18.98%), 3313 (19.25%), 3227 (19.47%), respectively (Table 2). According to the annotation results of KEGG database, the unigenes of three kinds of blueberries were associated with 129 metabolism pathways. The top five metabolism pathways were “Plant-pathogen interaction”, “Carbon metabolism”, “Ribosome”, “Protein processing in endoplasmic reticulum” and “Biosynthesis of amino acids” (S5 Table). GO enrichment analysis was used for functional annotation of unigenes, and 17,751, 18,237 and 17,503 unigenes hit 94,620, 97,611 and 94,168 GO terms with an average of 5.33, 5.35 and 5.38 hits per unigene (S6 Table). The “metabolic process” was the main term of “biological process” category, and “cell” and “cell part” terms were enriched in “cellular process” category, while “catalytic activity” and “binding” were significantly enriched in the “molecular function” category (S6 Table).

**Table 1:** Overview of unigenes annotation in transcriptome of three blueberry crops.

Database	Number (percentage) of total annotated unigenes		
	Rabbiteye blueberry	Southern highbush blueberry	Northern highbush blueberry
Nr	28028 (61.55%)	28029 (65.31%)	27189 (62.32%)
Swiss-Port	20510 (45.04%)	20753 (48.36%)	20084 (46.03%)
COG	17061 (37.475)	17213 (40.11%)	16570 (37.98%)
KEGG	10431 (22.91%)	10755 (25.06%)	10265 (23.51%)
Annotated by one or more above databases	28091 (61.69%)	28115 (65.51%)	27256 (62.47%)
None of the above four databases	17444 (38.31%)	14799 (34.49%)	16374 (37.53%)

**Table 2:** KOG (COG) annotation of unigenes in transcriptome of three blueberry crops.

Classification of molecular function	Number (percentage) of unigenes annotated by KOG		
	Rabbiteye blueberry	Southern highbush blueberry	Northern highbush blueberry
RNA processing and modification	1768 (%)	1729 (%)	1676 (%)
Chromatin structure and dynamics	469 (%)	502 (%)	464 (%)
Energy production and conversion	1060 (%)	1042 (%)	1026 (%)
Cell cycle control, cell division, chromosome partitioning	703 (%)	724 (%)	725 (%)
Amino acid transport and metabolism	754 (%)	792 (%)	776 (%)
Nucleotide transport and metabolism	222 (%)	232 (%)	228 (%)
Carbohydrate transport and metabolism	1054 (%)	1045 (%)	1007 (%)
Coenzyme transport and metabolism	188 (%)	202 (%)	194 (%)
Lipid transport and metabolism	897 (%)	910 (%)	868 (%)
Translation, ribosomal structure and biogenesis	1232 (%)	1269 (%)	1205 (%)
Transcription	1551 (%)	1615 (%)	1574 (%)
Replication, recombination and repair	889 (%)	885 (%)	894 (%)
Cell wall/membrane/envelope biogenesis	338 (%)	324 (%)	321 (%)
Cell motility	6 (%)	14 (%)	10 (%)
Posttranslational modification, protein turnover, chaperones	3238 (%)	3313 (%)	3227 (%)
Inorganic ion transport and metabolism	542 (%)	543 (%)	557 (%)
Secondary metabolites biosynthesis, transport and catabolism	840 (%)	857 (%)	798 (%)
General function prediction only	6204 (%)	6154 (%)	5990 (%)
Function unknown	1184 (%)	1206 (%)	1172 (%)
Signal transduction mechanisms	3451 (%)	3375 (%)	3311 (%)
Intracellular trafficking, secretion, and vesicular transport	1366 (%)	1444 (%)	1385 (%)
Defense mechanisms	195 (%)	208 (%)	190 (%)
Extracellular structures	73 (%)	68 (%)	80 (%)
Nuclear structure	105 (%)	108 (%)	94 (%)
Cytoskeleton	511 (%)	539 (%)	644 (%)

### Detection and statistics of R-Genes

We identified 3599, 3495 and 3513 candidate R-gene unigenes, which belong to more than 15 families in rabbiteye, southern highbush and northern highbush blueberries, respectively. The number of candidate R-gene families in three kinds of blueberries had almost the same trend. Candidate unigenes in RLP family has an absolute advantage in number, and reached to 996, 1055, 1016, which accounted for 27.67%, 30.19% and 28.92% of total candidate R-gene unigenes, followed by NL, N, CNL, TNL, and their numbers reached to 549 (15.25%), 509 (14.56%), 518 (14.75%); 504 (14.00%), 475 (13.59%), and 473 (13.46%); 417 (11.59%), 382 (10.93%), and 6 (11.56%); and 433 (12.03%), 374 (10.70%) and 397 (11.30%) in three blueberry crops, respectively (Table 3).

**Table 3:** Candidate R-gene identified from unigenes in the transcriptomes of three blueberry crops.

R-gene families	Number (percentage) of putative R-gene		
	Rabbiteye blueberry	Southern highbush blueberry	Northern highbush blueberry
RLP	996 (27.67%)	1055 (30.19%)	1016 (28.92%)
NL	549 (15.25%)	509 (14.56%)	518 (14.75%)
N	504 (14.00%)	475 (13.59%)	473 (13.46%)
TNL	417 (11.59%)	382 (10.93%)	406 (11.56%)
CNL	433 (12.03%)	374 (10.70%)	397 (11.30%)
RLK	252 (7.00%)	260 (7.44%)	270 (7.69%)
RLK-GNK2	141 (3.92%)	156 (4.46%)	138 (3.93%)
T	70 (1.94%)	67 (1.92%)	71 (2.02%)
CN	66 (1.83%)	63 (1.80%)	75 (2.13%)
Pto-like	44 (1.22%)	34 (0.97%)	34 (0.97%)
Mlo-like	22 (0.61%)	23 (0.66%)	18 (0.51%)
L	15 (0.42%)	15 (0.43%)	16 (0.46%)
RPW8-NL	6 (0.17%)	6 (0.17%)	6 (0.17%)
Other	84 (2.33%)	76 (2.17%)	75 (2.13%)
Total	3599	3495	3513

### Detection of SSR markers

We identified 8756, 9020, and 9198 SSR markers from 7251, 7282 and 7518 unigenes from rabbiteye, southern highbush and northern highbush blueberry cultivars. The numbers of SSR kinds with different core motifs exhibited similar distribution patterns in three blueberry crops, for example, two repeat motifs accounted for the majority in numbers, and reached to 5829, 6177, 6230, which accounted for 66.57%, 68.48% and 67.73% of total SSR markers in three blueberry crops, followed by 3, 4, 6 and 5 repeat type SSR motifs (Table 4). Of the all kinds of SSR markers with different motifs, “AG/CT” motif was found to be the highest proportion, which accounted for 61.96%, 63.80%, 62.85% of total SSR markers in three blueberry crops, followed by AAG/CTT motif which accounted for 8.0% of total SSR markers in three blueberry crops, while all other motifs accounted for less than 5% of total SSR markers in three blueberry crops. Most of the SSR markers were found to be suitable for sequence information to design primers (S7 Table).

**Table 4:** SSR markers identified from unigenes in transcriptome of three blueberry crops.

SSR motif	Number (percentage) of SSR markers		
	T1-Rabbiteye blueberry (8756)	T2-Southern highbush blueberry (9020)	T3-Northern highbush blueberry (9198)
AC/GT	313 (3.57%)	324 (3.59%)	324 (3.52%)
AG/CT	5425 (61.96%)	5755 (63.80%)	5781 (62.85%)
AT/AT	81 (0.94%)	89 (0.97%)	115 (1.25%)
AAC/GTT	113 (1.29%)	103 (1.14%)	99 (1.08%)
AAG/CTT	739 (8.44%)	774 (8.58%)	806 (8.76%)
AAT/ATT	27 (0.31%)	28 (0.31%)	32 (0.35%)
ACC/GGT	435 (4.97%)	382 (4.24%)	406 (4.41%)
ACG/CGT	114 (1.30%)	97 (1.08%)	97 (1.05%)
ACT/AGT	25 (0.29%)	25 (0.28%)	24 (0.26%)
AGC/CTG	294 (3.36%)	268 (2.97%)	286 (3.11%)
AGG/CCT	452 (5.16%)	414 (4.59%)	428 (4.65%)
ATC/ATG	172 (1.96%)	157 (1.74%	163 (1.77%)
CCG/CGG	129 (1.47%)	154 (1.71%)	156 (1.70%)
AAAG/CTTT	29 (0.33%)	28 (0.31%)	28 (0.30%)
AAAT/ATTT	32 (0.37%)	28 (0.31%)	34 (0.37%)
others	376 (4.29%)	394 (4.37%)	419 (4.55%)

### Identification of SNPs

**Fig 1:**
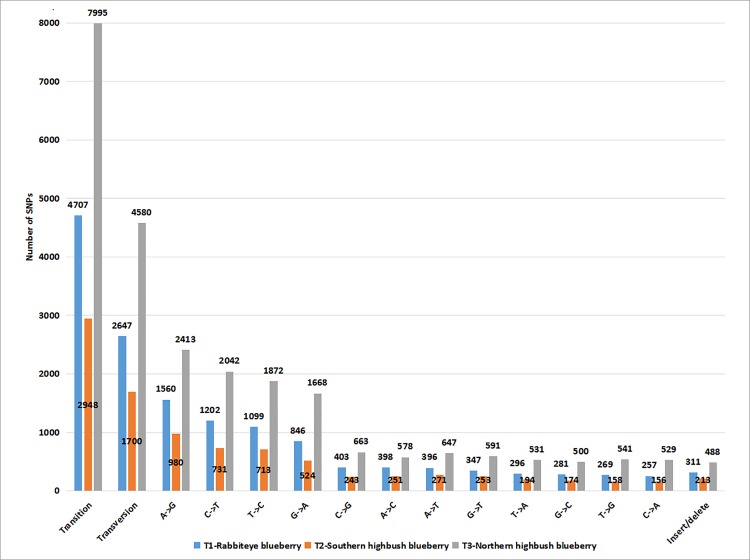
Statistics of SNP distribution pattern in transcriptome of three blueberry crops.

**Fig 2:**
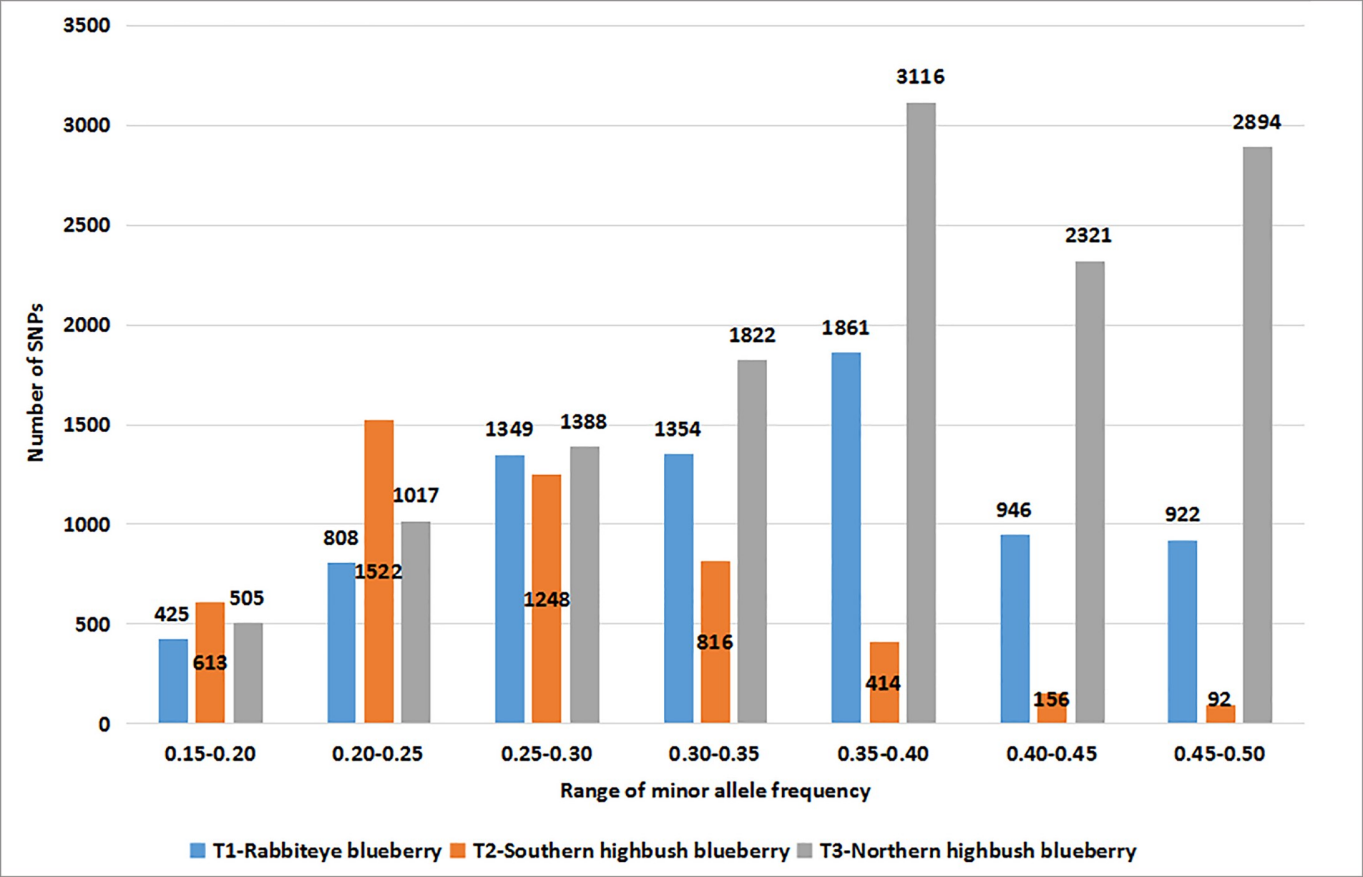
Minor allele frequency distribution of SNPs in transcriptome of three blueberry crops.

**Fig 3:**
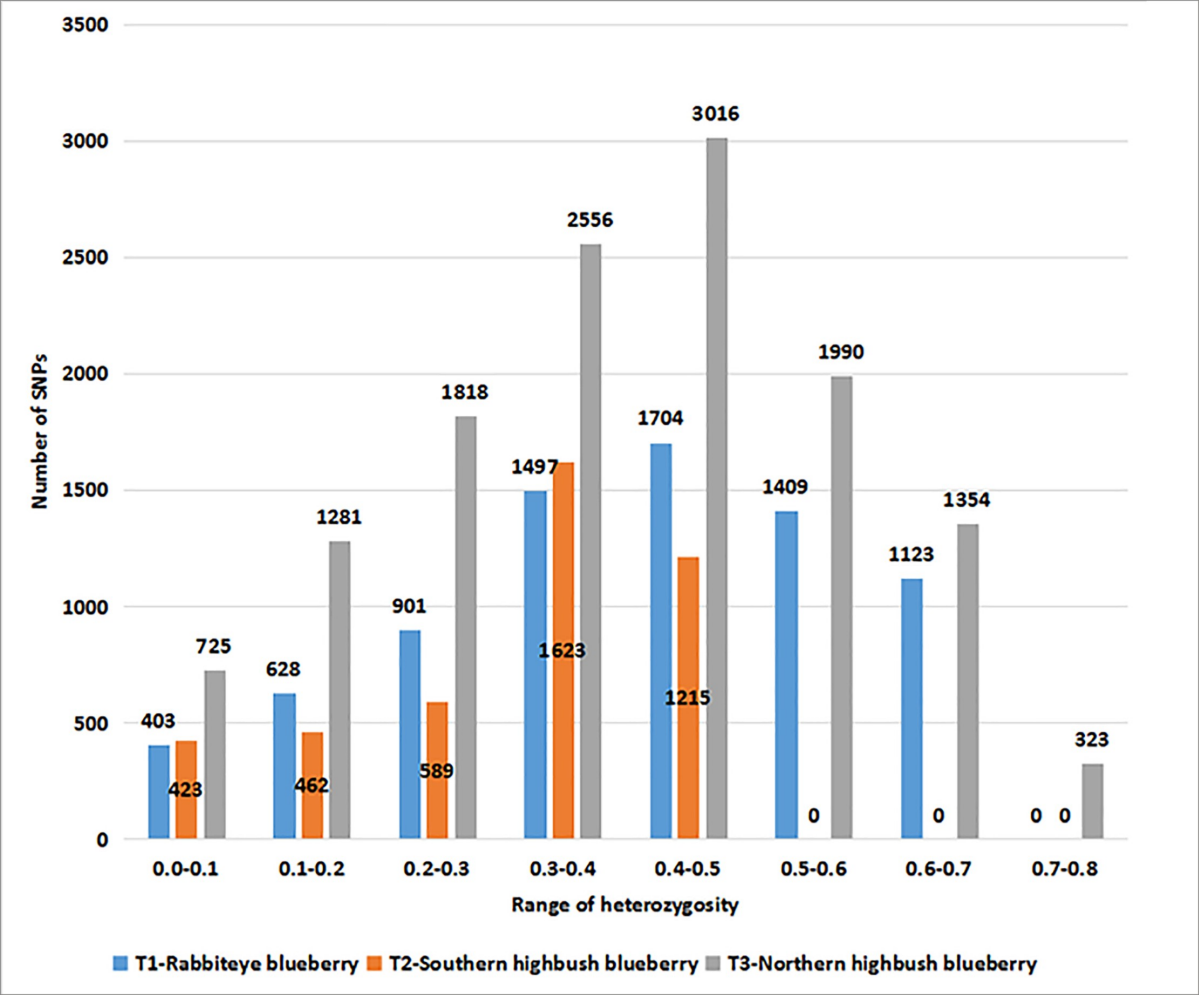
Heterozygosity distribution of SNPs in transcriptome of three blueberry crops.

After using a strict filtering procedure, we identified 7665, 4861, 13,063 SNPs in leaf`s transcriptome of rabbiteye, northern highbush and southern highbush blueberry cultivars, respectively (S8 Table). Among these SNPs, base mutants with transitions were 1.90, 1.93 and 1.93 times of transversion, and G/A, C/T mutant patterns were much higher than other mutants, and the numbers reached to 2647, 1770, 4580, and 1560, 980, 2413 that accounted for 34.53%, 36.41%, 35.06% and 20.35%, 20.16%, 18.47% of total SNPs in rabbiteye, northern highbush and southern highbush blueberry cultivars, respectively (Fig 1). The minor allele frequency of these SNPs were in the range of 0.15–0.50, and if we divided these values into seven intervals with 0.05 per interval, the minor allele frequency of most of the SNPs fall into 0.35–0.40 in rabbiteye and northern highbush blueberry, and 0.20–0.25 in southern highbush blueberry cultivars (Fig 2). The heterozygosity ratio of all the SNPs was in the range of 0.00–0.80 in three blueberry crops, and if we divided these heterozygosity values into 9 intervals with 0.10 per interval, most of the SNPs fall into the range of 0.4–0.5 in rabbiteye blueberry and northern highbush blueberry and 0.3–0.4 in southern highbush blueberry (Fig 3).

## Discussion

In the last decade, genomics research based on high-throughput sequencing for fruit crops had made a dramatic progress, and the reference genome of more than ten fruit crops and huge RNA data have been published. These investigations have greatly promoted the studies of molecular biology, evolution genetics and breeding program of fruit crops [52–55]. Recently, the genome of blueberry (*Vaccinium corymbosum* with diploid genome) has been published, and researchers can access it by the genome Browser8.5.2 software (http://bioviz.org/igb/). However, the assembly integrity and sequencing coverage of this reference genome is very low [36]. Therefore, we assembled the transcriptome with a method of no reference genome to get more information about gene functions in this study. In spite of a lot of genome information or transcriptome sequences have been deposited in Genebank [24–36], the reports on SSR or SNP markers at large scale are limited. In this study, we developed more than 8000 SSRs and 4000 high-quality SNPs markers in three kinds of blueberry crops based on the transcriptome data, and this would offer great help for the blueberry studies about molecular genetics, molecular breeding and association analysis that mainly rely on the molecular tools.

Plants have evolved a wide range of defense mechanisms to protect themselves against pathogens, and the major defense mechanisms are disease resistance which commonly mediated by semi-dominant or dominant R genes that encode receptors and detect pathogen infection either by recognition of pathogen effector molecules directly, or by recognition of effector modified host targets indirectly [56,57]. Crops are the plant groups that offer basic sources of energy and nutrition for human survival. There are number of factors that reduces the global crop yield, such as huge number of plants grown together, inadequate supply of fertilizer and water, and plants of a crop are more susceptible to a large number of pathogens, including bacteria, insects, oomycetes, and nematodes [58,59]. So, developing disease-resistant varieties by different methods, such as genetic transformation of plant resistance genes, are believed to be a good choice to protect crops from diseases, insects and pests. Identifying plant resistance genes and R-gene loci are the basic premise to assemble various resistance sources effectively and to engineer new strategies for disease resistance in agriculture [60,61]. In this study, we identified about thousands of unigenes that were homologous with R-gene that belong to more than 13 families, and this would offer the molecular information to understand the ecological adaption of blueberry. At the same time, these unigenes information also offer the basic molecular tools for resistance breeding.

In the past decade, single nucleotide polymorphisms (SNPs) have become a popular and conventional choice of genetic marker, especially for diploid species by high-throughput sequencing method [62–65]. However, identification of SNPs in polyploids is more challenging because of complex genome duplication events which incurring homologous SNPs (polymorphic positions occurring across subgenomes within and among individuals). SNP markers were produced at large scale by next generation sequencing platform in few polyploid species by using different methods to filter false positives [66]. For example, to filter out false positives as much as possible, the SNPs from uniquely mapping reads or the reads depth more than three have been used in transcriptome data of *B*. *napus* [67,68], and SNPs from these strategies have been successfully used for genome-wide association studies [69]. Another SNP filtering strategy was successfully used in potato by combining of read depth, quality and SNP density of transcriptome sequence [70]. Besides, a Network-Enabled Analysis Kit (UNEAK pipeline) implemented in the TASSEL-GBS software program (https://bitbucket.org/tasseladmin/tassel-5-source/wiki/Tassel5GBSv2Pipeline) has been developed and proven to be effective for the identification of SNPs in complex species such as switchgrass [71]. The conclusion drawn from above successful cases is that high-quality SNPs can be identified in even the most difficult polyploid species.

Most cultivated blueberry cultivars are polyploid, for example, lowbush blueberry is tetraploid [68], northern highbush blueberry is 2x, 4x and hexaploid (6x), and 3x and 5x are produced by hybridization [72,73], and rabbiteye blueberry is hexaploid [74]. The southern highbush and inter-highbush are also generated by crossing with northern highbush and other species, and both are polyploid [75]. To overcome the adverse effects incurred by complex genome duplication events, we further filtered out the original SNP dataset, which was generated by using program tophat v2.0.14 with default parameters. We systematically considered the status of SNP loci by sequencing quality, read depth, minor allele frequency, annotation statistics, and only from annotated single copy unigenes (a gene family includes only one unigene in a species). We believed that final SNP datasets are reliable molecular tools for the association studies and marker assisted breeding of blueberry.

## Supporting information

S1 Table Raw reads statistics of transcriptome in three blueberry crops. (XLSX)

S2 Table Unigenes assembled information of transcriptome in three blueberry crops. (XLSX)

S3 Table Species distribution of Nr annotation of unigenes in transcriptome of three blueberry crops. (XLSX)

S4 Table E-value distribution of Swiss-Port annotation of unigenes in transcriptome of three blueberry crops. (XLSX)

S5 Table KEGG annotation of unigenes in three blueberry crops. (XLS)

S6 Table GO enrichment analysis of unigenes identified in three blueberry crops. (XLS)

S7 Table SSR loci identified and their corresponding primers designed in the unigenes of three blueberry crops. (XLSX)

S8 Table SNPs loci identified from leaf transcriptome of three blueberry crops. (XLSX)
